# The Protective Effects of *Polygala tenuifolia* and Tenuifolin on Corticosterone-Evoked Ferroptosis, Oxidative Stress, and Neuroinflammation: Insights from Molecular Dynamics Simulations and In Vitro Experiments

**DOI:** 10.3390/foods13213358

**Published:** 2024-10-23

**Authors:** Chaoyi Xue, Zhiyong He, Maomao Zeng, Zhaojun Wang, Qiuming Chen, Fang Qin, Mingmin Chen, Hui Ye, Jie Chen

**Affiliations:** 1 School of Food Science and Technology, Jiangnan University, Wuxi 214122, China; 7210112108@stu.jiangnan.edu.cn (C.X.); zyhe@jiangnan.edu.cn (Z.H.); mmzeng@jiangnan.edu.cn (M.Z.); zhaojun.wang@jiangnan.edu.cn (Z.W.); chenqm@jiangnan.edu.cn (Q.C.); qfflast@sina.com (F.Q.); 2 State Key Laboratory of Food Science and Technology, Jiangnan University, Wuxi 214122, China; 3 School of Chemistry, Chemical Engineering and Biotechnology, Nanyang Technological University, Singapore 637371, Singapore; chen1888@ntu.edu.sg

**Keywords:** tenuifolin, ferroptosis, oxidative stress, neuroinflammation, corticosterone, molecular dynamics simulation

## Abstract

Excessive stress is a well-established contributor to neurological damage, insomnia, and depression, imposing a significant burden on individuals and society. This underscores the urgent need for effective stress-relief strategies. The main purpose of this study was to explore the protective effects of *Polygala tenuifolia* (PT) and its bioactive compound, tenuifolin, against corticosterone-induced neurotoxicity, with a focus on ferroptosis, oxidative stress, and neuroinflammation. Both PT extracts and tenuifolin mitigated corticosterone-induced cellular damage. Tenuifolin reversed the corticosterone-induced dysregulation of ferroptosis-associated proteins, such as SLC7A11, GPX4, and Nrf2, leading to a marked reduction in ferroptosis levels. Molecular dynamics simulations revealed that corticosterone significantly altered the conformation and binding energy of the SLC7A11/SLC3A2 complex, critical for ferroptosis regulation. These changes were reversed by tenuifolin. Additionally, tenuifolin alleviated corticosterone-induced oxidative stress and neuroinflammation, both of which accelerated ferroptosis. In conclusion, these results indicate that tenuifolin attenuates corticosterone-induced neurotoxicity by modulating ferroptosis, oxidative stress, and neuroinflammation. This study provides a theoretical foundation for the application of PT and tenuifolin in stress-induced nerve damage.

## 1. Introduction

With increased competition and the rising cost of living, individuals are facing increased work-related and psychological stress [1]. Long-term stress can bring about abnormal activation of the hypothalamic–pituitary–adrenal (HPA) axis, triggering an excessive release of corticosterone, which may cause nerve damage [2]. This damage is closely associated with conditions such as depression, sleep disorders, anxiety, and cognitive decline. These conditions not only diminish the quality of life but also cause heavy financial strain [3]. Hence, exploring effective treatments for stress-induced nerve damage and related mental disorders has become a cutting-edge topic in neuroscience.

Ferroptosis, a newly identified form of cell death, involves the disruption of intracellular iron metabolism and the accumulation of reactive oxygen species (ROS), leading to excessive lipid peroxidation [4]. This process is driven by imbalances in iron metabolism and the depletion of glutathione (GSH). Iron ions (Fe^2+^) act as catalysts in lipid peroxidation, while glutathione peroxidase 4 (GPX4), a principal antioxidant defense enzyme, protects cells from lipid peroxides. When the activity of GPX4 is reduced, cells struggle to remove peroxidized lipids, which cause cell damage and death. Ferroptosis is regulated through several pathways, including those involving nuclear factor erythroid 2-related factor 2 (NRF2), ferritin, transferrin, and the transferrin receptor. In animal models of depression, exacerbated symptoms are linked with elevated ferroptosis, suggesting that ferroptosis may promote this process, worsening nerve damage and depressive symptoms [5]. Iron metabolism imbalance, elevated iron ferroptosis protein expression, impaired GSH metabolism, and lipid peroxidation were significantly associated with Alzheimer’s disease [6]. Enhanced iron concentration in serum was found in 5xFAD mice, accompanied by emotional and cognitive impairment and significant neurodegeneration [7,8,9]. Therefore, targeting ferroptosis presents a potential therapeutic strategy for stress-induced nerve damage.

The heterodimeric complex of SLC7A11/SLC3A2 plays a pivotal role in ferroptosis [10]. This complex facilitates the intracellular transport of cystine, which is necessary for GSH synthesis. GSH is crucial for producing GPX4, a key enzyme responsible for detoxifying harmful lipid peroxides. Consequently, the SLC7A11/SLC3A2 complex is a core target in the regulatory mechanisms of ferroptosis [11]. Although the role of SLC7A11/SLC3A2 in ferroptosis is widely acknowledged, there is limited research on how corticosterone and its antagonists affect this complex under stress conditions. This gap in knowledge calls for further investigation.

The association between neuroinflammation and stress-induced nerve damage has emerged as a key area of research [12]. Neuroinflammatory responses in the central nervous system (CNS) are characterized by microglial activation, the production of pro-inflammatory cytokines, and the disruption of the blood–brain barrier (BBB). Under pathological conditions such as stress, infection, or neurodegenerative diseases, pro-inflammatory cytokines, including tumor necrosis factor alpha (TNF-α), interleukin-1 beta (IL-1β), and interleukin-6 (IL-6), are released in excess [13]. Chronic stress raises corticosterone levels, worsening neuroinflammation and stress-related damage [14]. Thus, we are encouraged to explore targeting neuroinflammation as a novel approach to treating stress-related damage.

PT, a traditional medicinal plant, has long been used to improve cognitive function, reduce insomnia, ameliorate memory decline, and modulate affective disorders, such as depression [15]. Research has found that the PT extract had significant anti-inflammatory effects in the LPS mouse model [16]. Triterpenoid saponins from PT were shown to reduce the release of the inflammatory cytokine TNF-α in LPS-stimulated BV2 cells [17]. In the PD model, PT significantly ameliorated dopaminergic neuronal degeneration and inhibited activation of NLRP3 inflammatory vesicles in the substantia nigra, alleviating the PD phenotype [18]. PT ameliorated cognitive dysfunction, oxidative stress, and tau hyperphosphorylation in AD models [19].

The constituents of PT extract have been explored by previous studies and mainly include saponins, xanthones, oligosaccharide esters, alkaloids, organic acids, flavonoids, etc. Studies have revealed that its active constituent, tenuifolin (approximately 2% of PT) [20], can prevent nerve cell death in neurodegenerative diseases like Alzheimer’s and Parkinson’s [21]. Recent research has demonstrated that tenuifolin improved cognitive deficits induced by chronic restraint stress in C57BL/6J mice by reducing the secretion of neuroinflammatory cytokines and corticosterone [22]. It could also prevent AD-like phenotypes through multiple signaling by inhibiting oxidative stress, maintaining stability of the calpain system, and inhibiting neuronal apoptosis [23].

The above studies illustrated that PT and tenuifolin have a strong neuroprotective capacity with significant anti-oxidative stress and anti-inflammatory effects, and these two pathways are closely related to ferroptosis. Considering the pivotal roles of ferroptosis, oxidative stress, and neuroinflammation in stress-induced nerve damage and given the established protective effects of tenuifolin, this study hypothesizes that PT and tenuifolin may reduce corticosterone-induced nerve damage by inhibiting ferroptosis, neuroinflammation, and oxidative stress. The corticosterone-treated PC12 cell line serves as a model for investigating the neuroprotective effects of PT and tenuifolin, as well as their influence on ferroptotic signaling pathways, neuroinflammation, and oxidative stress levels. Furthermore, molecular dynamics simulations will be employed to examine the potential effects of corticosterone and tenuifolin on the structure and function of the SLC7A11/SLC3A2 complex.

## 2. Materials and Methods

### 2.1. Materials

The PC12 cell line (Chinese Academy of Sciences’ Cell Bank, Shanghai, China). Fetal bovine serum (FBS), horse serum, and RPMI 1640 medium (Gibco Laboratory Co., Ltd., Grand Island, NY, USA). Corticosterone, cell counting kit-8, ROS kit, and additional supplies (Beyotime Co., Ltd., Shanghai, China). Antibodies specific to SLC7A11, GPX4, Nrf2, and other targets (Proteintech Co., Ltd., Wuhan, China). PT was acquired from a local market. Tenuifolin (Yuanye Bio-Technology Co., Ltd., Shanghai, China). All other utilized chemicals and reagents were of the highest analytical quality.

### 2.2. Cell Culture and Treatment

PC12 cell cultures were maintained in RPMI 1640 medium supplemented with 10% FBS, 100 μg/mL streptomycin, and 100 IU/mL penicillin at a temperature of 37 °C in a humidified environment containing 5% CO_2_. Prior to exposure to corticosterone for 12 h, the cells were pre-incubated with either PT ethanol extract (PTE) or tenuifolin for 12 h, followed by a PBS rinse to remove any residual treatment. The culture medium was refreshed daily. Subsequently, the cells were harvested for further analysis.

### 2.3. Cell Viability Assay

PC12 cells were cultured in 96-well plates (NEST Biotechnology Co., Ltd., Wuxi, China). Once the medium was aspirated, the cells were subjected to a 12-hour treatment with either PT extract or tenuifolin, followed by a 12-hour exposure to corticosterone. The method for preparing the PTE was in accordance with previously published procedures; the root of the PT was used [24]. Subsequently, 10 μL of CCK-8 reagent was introduced into each well, and the cells were further incubated at 37 °C for 2 h. The optical density (OD) at 450 nm was then measured, with each experimental condition being replicated six times.

### 2.4. Determination of Iron Ions, MDA, Inflammatory Factors, ROS, GPX, SOD, and GSH Activity

Concentrations of iron ions, malondialdehyde (MDA), and inflammatory cytokines (IL-1β, IL-6, and TNF-α) were determined using assay kits from Elabscience, China. ROS levels were assessed with the DCFH-DA probe (Beyotime Co., Ltd., Shanghai, China). For this, PC12 cells were treated with DCFH-DA at 37 °C for 30 min, after which the fluorescence was measured using a fluorescence spectrophotometer. Activities of glutathione peroxidase (GPX), superoxide dismutase (SOD), and GSH were quantified using kits supplied by Beyotime Co., Ltd., Shanghai, China.

### 2.5. Western Blotting

Protein detection in PC12 cells followed protocols outlined in a prior publication [25]. Cells, post-treatment, were lysed with a lysis buffer from Beyotime, China, to extract proteins. Protein concentrations were measured with a BCA kit (Beyotime Co., Ltd., Shanghai, China). The lysates were mixed with SDS sample buffer and denatured at 100 °C for 10 min. Each 20 μg protein sample was separated by SDS-PAGE gels and then transferred onto PVDF membranes. After blocking the membranes with milk for 1 h, they were incubated with primary antibodies at 4 °C overnight. Following this, the membranes were treated with secondary antibodies, and the protein bands were visualized and quantified using a chemiluminescent detection system.

### 2.6. Immunofluorescence Analysis

Following tenuifolin treatment, cells were cultured with corticosterone at 37 °C in a 5% CO_2_ incubator. Subsequently, cells were rinsed with PBST and fixed using a 4% paraformaldehyde solution for 15 min. After another PBST wash, the cells were permeabilized with 0.1% Triton X-100 (Beyotime Co., Ltd., Shanghai, China) for 30 min. Once washed and blocked, the cells were incubated with primary antibodies at 4 °C overnight. The next day, cells were incubated with a secondary antibody at room temperature (24 °C) for 1 h and then stained with 4′,6-diamidino-2-phenylindole dilactate for 1 min. The cells were finally examined using a confocal microscope.

### 2.7. Molecular Docking and Molecular Dynamics Simulations

The 3D model of the SLC7A11/SLC3A2 complex, with the PDB identifier 7P9V, was retrieved from the RCSB Protein Data Bank(rcsb.org) funded by US. The molecular structures of corticosterone and tenuifolin were sourced from the PubChem repository. These structures were then refined using the b97-3C method in the ORCA 5.0 software suite [26], after which molecular docking analysis was performed. Initially, polar hydrogen atoms and Gasteiger charges were added to both the protein and the ligands. The AutoDock Tools 4.2 was utilized to merge non-polar hydrogen atoms. The docking analysis was initiated with AutoDock Vina [27], which conducted a global search across the entire protein to pinpoint the optimal binding site. During the docking process, all rotatable bonds of both corticosterone and tenuifolin were allowed to rotate freely.

To delve deeper into the binding stability of the protein with corticosterone and tenuifolin, a 50-nanosecond molecular dynamics (MD) simulation was executed using Gromacs 5 [28] on the optimized docked structures. The simulation box, a cube with a 10 Å clearance from the protein surface, was solvated using the SPC water model. The protein’s topology was set up with the AMBER99SB-ILDN force field [29], and Na^+^ and Cl^−^ ions were added to ensure charge neutrality. Before starting the MD simulations, energy minimization was carried out using the steepest descent method. The protein–ligand complex was then equilibrated for 2 ns in the NVT ensemble and 3 ns in the NPT ensemble, using the Berendsen temperature and Parrinello–Rahman pressure controls to stabilize at 25 °C and 1 bar, respectively. After equilibration, a 50-nanosecond MD simulation was run. The structural analysis involved calculating RMSD, RMSF, Rg, and hydrogen bond parameters. The thermodynamic stability of corticosterone and tenuifolin in the binding sites was evaluated using MMPBSA calculations, with residue contributions in the binding pockets analyzed by the gmx_mmpbsa utility [30].

### 2.8. Statistical Analysis

Results are expressed as mean values ± standard deviations, derived from a minimum of three separate experiments. Statistical significance between control and experimental groups was determined using one-way ANOVA, followed by Tukey’s post-hoc test with SPSS16.0 software. A threshold of *p* < 0.05 was set for statistical significance across all studies. Graphs were created using GraphPad 8.0 and Figdraw (www.figdraw.com).

## 3. Results and Discussion

### 3.1. PTE and Tenuifolin Protected PC12 Cells from Corticosterone-Induced Cytotoxicity

**Figure 1:**
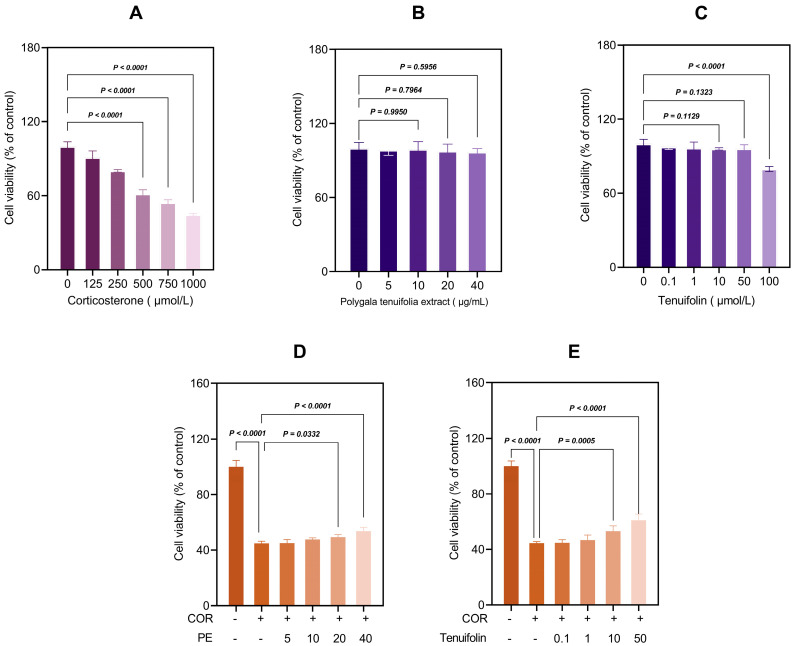
Tenuifolin protected PC12 cells from corticosterone−induced cytotoxicity. PC12 cells were treated with (**A**) different concentrations of corticosterone (0, 125, 250, 500, 1000 μM) for 12 h or (**B**,**C**) different concentrations of PTE (5, 10, 20, 50 μg/mL) and tenuifolin (0, 0.1, 1, 10, 50, 100 μM) for 12 h, and then the cell viability was measured by CCK−8 assay. (**D**,**E**) PC12 cells were pre−treated with different concentrations of PTE and tenuifolin for 12 h and then with corticosterone (750 μM) for 12 h, and then the cell viability was measured by CCK−8 assay. Data are presented as the mean ± SD, *n* = 6.

We studied the protective effects of PTE and tenuifolin against corticosterone-induced damage in PC12 cells. As shown in Figure 1, corticosterone decreased cell viability in a dose-dependent manner, e.g., 750 μM of corticosterone decreased the viability to 44.60% (*p* < 0.01, Figure 1A), but PTE (ranging from 5–40 μg/mL) and tenuifolin at (0.1–50 μM) did not show any toxic effects in the PC12 cells (Figure 1B,C). To assess the protective effect of tenuifolin, PC12 cells were pre-treated with PTE (5–40 μg/mL) or tenuifolin (0.1–50 μM) for 12 h before exposure to 750 μM corticosterone for 12 h. Both PTE and tenuifolin significantly improved cell viability in a dose-dependent manner compared to the corticosterone-only group (Figure 1D,E). These findings highlight the significant protective role of tenuifolin in preventing corticosterone-induced neuronal damage.

These results elucidated the significant neuroprotective effects of tenuifolin against corticosterone-induced neuronal damage. Previous studies have shown that tenuifolin improves cognitive deficits in C57BL/6J mice under chronic restraint stress by lowering neuroinflammatory markers and corticosterone levels [22]. However, the specific effects of tenuifolin on corticosterone-induced neuronal damage were not well studied. Our findings fill this gap, revealing that tenuifolin markedly reduces the harmful impacts of corticosterone on neuronal health. Thus, it is likely that tenuifolin could reduce stress-induced nerve damage and related behavioral issues by decreasing corticosterone levels and limiting its neurotoxic effects. Tenuifolin at concentrations of 1, 10, and 50 μM showed the best protective effects on PC12 cells, increasing cell survival to 46.84%, 53.19%, and 61.01%, respectively.

### 3.2. Tenuifolin Inhibited Ferroptosis-Related Protein Expression in PC12 Cells Exposed to Corticosterone

**Figure 2:**
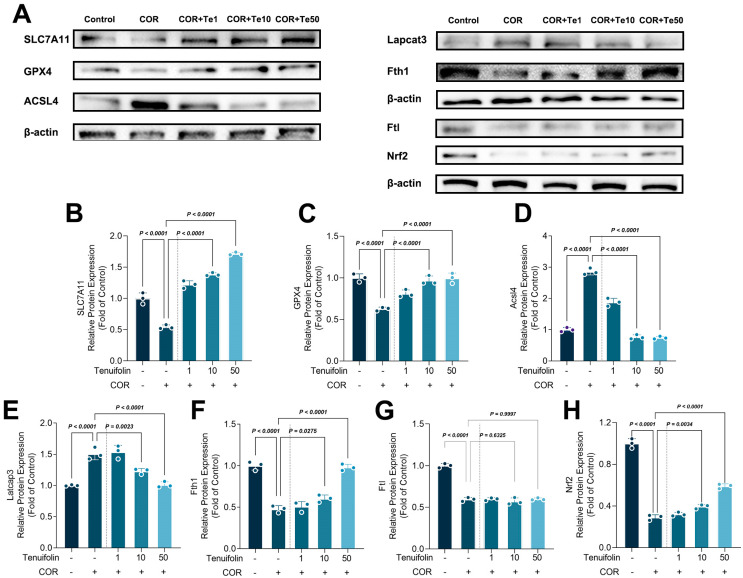
Tenuifolin inhibited ferroptosis−related protein expression in PC12 cells exposed to corticosterone. PC12 cells were treated with different concentrations of tenuifolin (1, 10, and 50 μM) and then corticosterone, and expressions of SLC7A11, GPX4, Acsl4, Lpcat3, Ftl, Fth1, and Nrf2 were measured by western blotting (**A**). Quantification of the blots was shown in (**B**–**H**). Data are presented as the mean ± SD, *n* = 3.

Ferroptosis, characterized by the occurrence of iron-dependent lipid peroxidation, plays a vital role in stress-induced neuron damage [31]. Figure 2 illustrates the ferroptosis levels in PC12 cells under corticosterone and tenuifolin treatment. PC12 cells treated with corticosterone exhibited lower SLC7A11 and GPX4 expressions, but tenuifolin reversed this effect (Figure 2B,C). Acsl4 and Lpcat3 levels were elevated with corticosterone treatment, while Fth1, Ftl, and Nrf2 levels were reduced. Tenuifolin reversed most of these changes, except for Ftl. Isoforsythiaside suppressed ferroptosis and neuroinflammation in erastin-impaired HT22 cells and APP/PS1 mice, and it also elevated the expression levels of FTL [32]. Forsythoside A administration alleviated the Alzheimer’s-like pathology by inhibiting ferroptosis-mediated neuroinflammation in the brains of APP/PS1 mice, with an upregulation of FTL [33]. This study did not observe an intervention of TE on the expression of FTL, possibly due to the use of corticosterone to treat PC12 cells to create a depression model, which differs from the Alzheimer’s disease model used in the aforementioned studies.

Acsl4 and Lpcat3 are involved in the synthesis of PUFA-PL, which is prone to peroxidation and can promote ferroptosis [34]. Reducing the expression of Acsl4 and Lpcat3 could lower ferroptosis [35]. The results showed that corticosterone and tenuifolin can interfere with the ferroptosis process by promoting or inhibiting Acsl4 and Lpcat3 expression (Figure 2A,D,E). Ftl and Fth1 control iron storage, and corticosterone reduces Fth1 expression, which in turn reduces iron storage, leading to iron overload in cells and promoting the occurrence of ferroptosis. Nrf2 is overexpressed during oxidative stress to activate antioxidant gene expression, but corticosterone promotes ferroptosis by inhibiting Nrf2 expression, thereby increasing oxidative stress levels.

**Figure 3:**
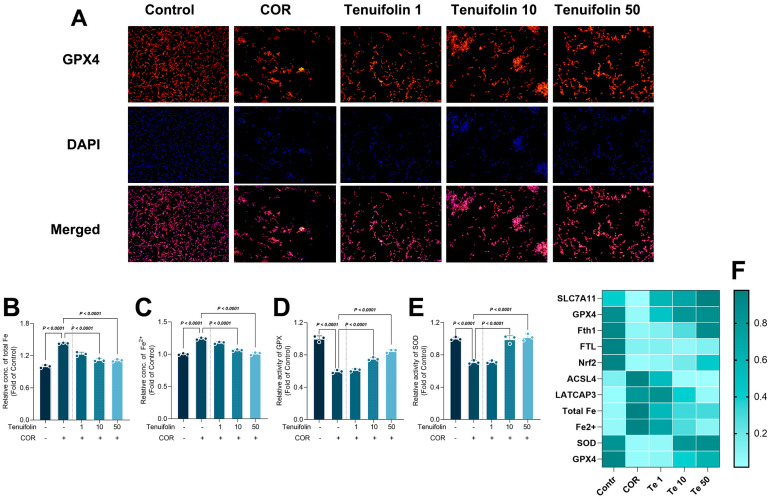
Tenuifolin inhibited ferroptosis in PC12 cells exposed to corticosterone. PC12 cells were treated with different concentrations of tenuifolin (1, 10, and 50 μM) and then corticosterone, and immunofluorescence staining of GPX4 was conducted (**A**) (Orange labels the GPX4 protein, blue labels the nucleus, pink is the merging of the two graphs). Total iron, Fe^2+^, and activity of SOD and GPX (**B**–**F**). Data are presented as the mean ± SD, *n* = 3.

The levels of total iron, Fe^2+^, and activity of SOD and GPX in the cell were also detected (Figure 3). Corticosterone treatment significantly increased total iron and Fe^2+^ levels in PC12 cells, but tenuifolin reduced them (Figure 3B). The change in Fe^2+^ levels is the opposite of Fth1, suggesting that corticosterone and tenuifolin can regulate ferroptosis by influencing Fe^2+^ levels. The activities of SOD and GPX also followed the pattern of Nrf2, indicating that corticosterone and tenuifolin can regulate antioxidant enzymes to affect ferroptosis and cell fate.

In animal models of depression or anxiety, increased corticosterone expression is often accompanied by elevated ferroptosis. Certain compounds, such as Xiaoyao San and edaravone, have been shown to reduce anxiety and depressive behavior by inhibiting ferroptosis-related pathway activation [36]. Similarly, in our study, corticosterone treatment increased ferroptosis levels, while tenuifolin reduced both corticosterone-induced cell damage and ferroptosis.

### 3.3. Tenuifolin Attenuated Structural Change in the SCL7A11/SCL3A2 Complex

**Figure 4:**
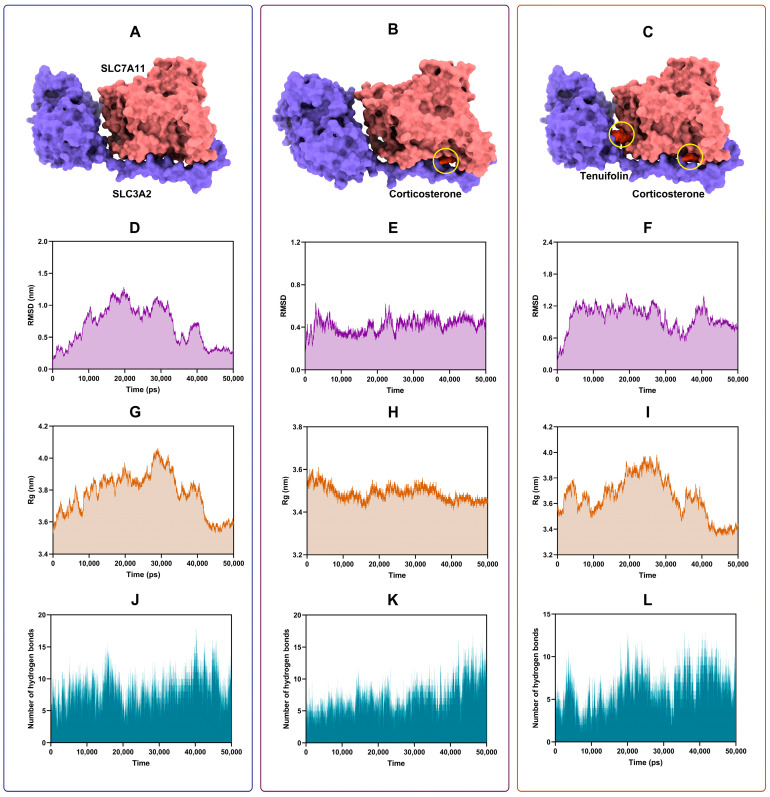
Molecular docking and molecular dynamics simulation results of the SCL7A11/SCL3A2 complex with corticosterone and tenuifolin. (**A**–**C**) Molecular docking conformation of the SCL7A11/SCL3A2 complex with corticosterone and tenuifolin. (**D**–**F**) RMSD results of the molecular dynamics simulation. (**G**–**I**) Rg results. (**J**–**L**) Hydrogen bond number results.

Computational molecular simulation techniques were employed to elucidate the potential complex interactions between proteins and ligands. The binding modes and affinities between small molecules and receptors can be preliminarily determined through molecular docking [37]. The docking result revealed that both corticosterone and tenuifolin could stably bind to the SCL7A11/SCL3A2 complex (Figure 4A–C), with binding energies of −7.74 and −7.96 kcal/mol, respectively. Multiple types of interactions, such as hydrogen bonds, van der Waals forces, or electrostatic interactions, may have contributed to the formation of these stable complexes.

To further validate these findings and compare the changes between the SCL7A11/SCL3A2 complex and the ligands (corticosterone and tenuifolin), a 50 ns MD simulation was conducted. This allowed us to observe how the protein–ligand complexes change over time. The root-mean-square deviation (RMSD) of protein atomic skeletons was used to analyze the positional shifts and conformational fluctuations of protein–ligand complexes during the simulation [38]. The RMSD plot (Figure 4D–F) shows that the curves gradually stabilized by the end of the simulation, indicating that the conformation of the complexes formed by the protein and the ligand became stable.

The radius of gyration (Rg) was used to determine the overall compactness of the complex structures [39]. The Rg remained stable throughout the simulation, especially in the later stages (Figure 4G–I), suggesting that the conformation of the complexes no longer underwent significant changes. Additionally, the number of hydrogen bonds between the ligands and the proteins in the complex is shown in Figure 4J–L. Stable hydrogen bonds were formed throughout the entire MD simulation process, with the number of hydrogen bonds ranging from 5 to 15. The formation of hydrogen bonds greatly contributes to the stability of the complex structure [40]. Overall, the results indicated that the SCL7A11 and SCL3A2, as well as their ligands—corticosterone and tenuifolin—formed stable structures.

**Figure 5:**
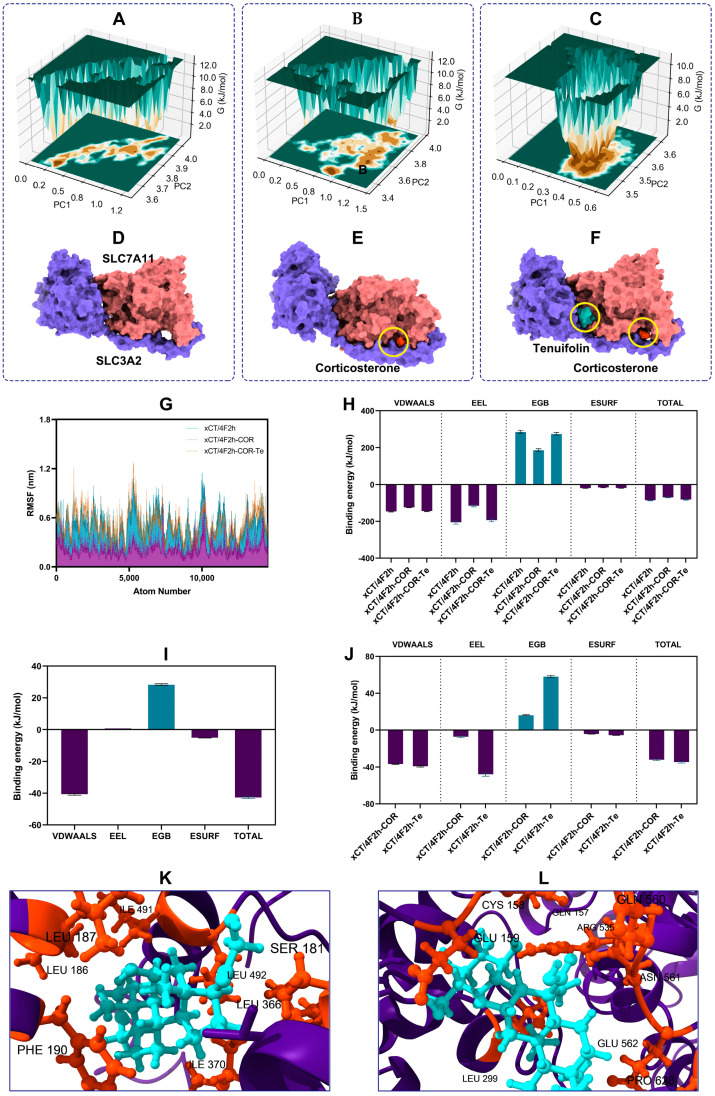
Molecular dynamics simulation results of the SCL7A11/SCL3A2 complex with corticosterone and tenuifolin. (**A**–**C**) Free energy landscape of the SCL7A11/SCL3A2 complex with corticosterone and tenuifolin. (**D**–**F**) Molecular dynamics simulation conformation with lowest energy. (**G**) RMSF results. (**H**) Binding energy between SCL7A11 and SCL3A2. (**I**,**J**) Binding energy between the SCL7A11/SCL3A2 complex and corticosterone/tenuifolin. (**K**,**L**) Binding site between the SCL7A11/SCL3A2 complex and corticosterone/tenuifolin.

Free energy landscape maps were generated to identify the energy minima (Figure 5A–C). The lowest energy conformations are shown in Figure 5D–F. At the lowest energy, the ligand remains within the protein receptor cavity, indicating stable binding. Corticosterone induced significant conformational changes in the SCL7A11/SCL3A2 complex, but tenuifolin markedly mitigated these changes.

The root mean square fluctuation (RMSF) was analyzed to represent the root mean square value of the average positional fluctuations of atoms or residues during the simulation process [41]. Higher RMSF values indicate more flexibility in parts of the molecule [42]. Figure 5G shows corticosterone changed the fluctuation pattern in the SCL7A11/SCL3A2 complex, while tenuifolin markedly alleviated this change, indicating that tenuifolin can mitigate the conformational alterations induced by corticosterone in the complex.

The binding energies between SCL7A11 and SCL3A2 were calculated before and after the addition of corticosterone and tenuifolin. As shown in Figure 5H, van der Waals interactions and electrostatic forces were the main forces keeping the SCL7A11 and SCL3A2 together in a complex. Corticosterone weakened these forces, but tenuifolin enhanced them. These findings align with the RMSF results, further supporting that tenuifolin prevented the conformational changes in the complex induced by corticosterone.

Furthermore, we also evaluated the binding characteristics of corticosterone and tenuifolin with the SCL7A11/SCL3A2 complex. When corticosterone binds to the SCL7A11/SCL3A2 complex alone, van der Waals interactions and nonpolar solvation energy were the primary binding forces. However, when tenuifolin was added, electrostatic forces also facilitated the binding of corticosterone to the SCL7A11/SCL3A2 complex. The introduction of tenuifolin reduced the total binding energy of corticosterone with the SCL7A11/SCL3A2 complex, indicating that tenuifolin can alleviate the impact of corticosterone on the SCL7A11/SCL3A2 complex. In addition, in the binding of tenuifolin to the SCL7A11/SCL3A2 complex, electrostatic forces, van der Waals interactions, and nonpolar solvation energy were the main binding forces, with electrostatic forces being the most critical. Residue energy decomposition analysis revealed that amino acids such as GLU:493, LEU:187, ILE:370, LEU:186, and ARG:183 play a significant role in the binding process of corticosterone with the complex (Figure 5K,L). For tenuifolin, the key residues include GLU:159, LEU:299, GLN:157, GLU:562, and ASN:561, which are critical for binding to the complex.

### 3.4. Tenuifolin Attenuated Corticosterone-Induced Oxidative Stress and Neuroinflammation in PC12 Cells

During stress-induced nerve damage, prolonged high levels of corticosterone can cause a series of adverse effects on the organism, including impacting the status of oxidative stress [43]. Oxidative stress occurs when ROS production exceeds the capacity of the antioxidant defense system, leading to damage to lipids, proteins, and nucleic acids. Chronic corticosterone can disrupt mitochondrial function, increasing ROS levels and triggering oxidative stress [44]. It can also reduce the activity of antioxidant enzymes, such as SOD and GPX, reducing the antioxidant capacity [45]. Elevated ROS levels can induce lipid peroxidation in cell membranes, producing harmful products such as MDA, which can further damage cellular structure and function.

**Figure 6:**
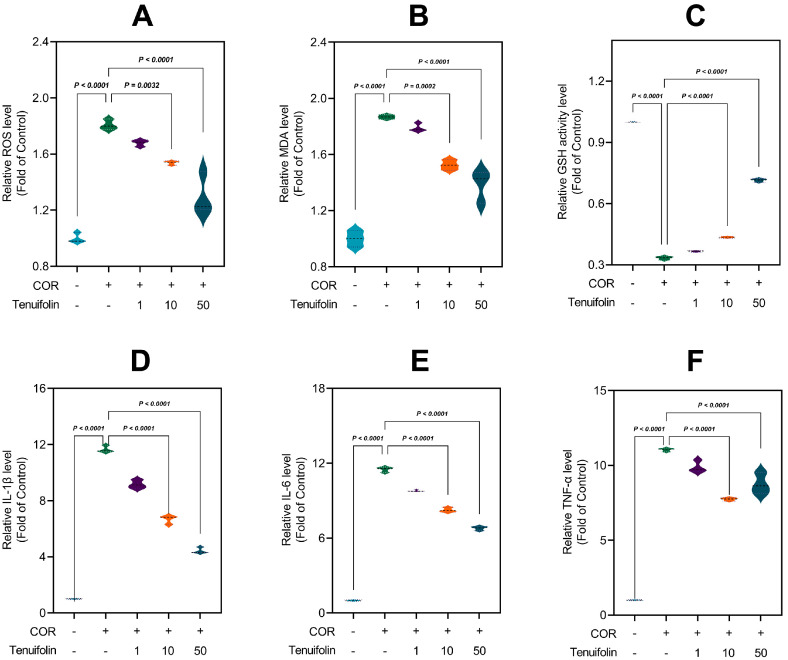
Tenuifolin alleviated oxidative stress and neuroinflammation in PC12 cells after corticosterone treatment. PC12 cells were treated with different concentrations of tenuifolin (1, 10, and 50 μM) and then corticosterone. (**A**) ROS level. (**B**) MDA level. (**C**) GSH activity level. (**D**–**F**) IL-1β, IL-6, and TNF-α level. Data are presented as the mean ± SD, n = 3.

To assess oxidative stress, we measured the levels of ROS. Corticosterone significantly increased ROS levels to 1.81-fold higher than the control group (Figure 6A), consistent with another study [46]. Treatment with tenuifolin at low, medium, and high doses reduced ROS levels to 1.68-fold, 1.54-fold, and 1.3-fold (Figure 6A), respectively, indicating a dose-dependent reduction in corticosterone-induced ROS production. The content of MDA was also measured to evaluate the degree of lipid peroxidation. Corticosterone treatment significantly elevated MDA content to 1.87-fold of the control, as found in a previous study [45]. After tenuifolin treatment at low, medium, and high doses, MDA levels reduced to 1.79-fold, 1.53-fold, and 1.38-fold, respectively (Figure 6B). Lastly, we measured the content of GSH to assess changes in the cellular antioxidant capacity. Corticosterone treatment decreased GSH content to 0.33-fold of the control. Tenuifolin treatment with low, medium, and high doses restored them to 0.37-fold, 0.43-fold, and 0.71-fold (Figure 6C), respectively.

Neuroinflammation is another key factor in stress-induced nerve damage. High corticosterone levels can activate microglia and promote the release of inflammatory cytokines, which can worsen neuroinflammation and directly damage neurons, potentially leading to apoptosis. Neuroinflammation can regulate cell survival and death by affecting intracellular signaling pathways, such as nuclear factor kappa B (NF-κB) and mitogen-activated protein kinases (MAPK) [47].

To assess the extent of inflammatory responses, we measured the expression levels of IL-1β, an inflammatory marker. Corticosterone treatment significantly increased IL-1β expression levels to 11.66-fold of the control, similar to a previous study [48]. Tenuifolin treatment reduced IL-1β expression levels to 9.17-fold, 6.66-fold, and 4.44-fold (Figure 6D), respectively, depending on the dose. We also measured the expression levels of IL-6. Corticosterone treatment significantly elevated IL-6 expression levels to 11.5-fold, while tenuifolin treatment reduced them to 9.81-fold, 8.25-fold, and 6.79-fold (Figure 6E), respectively, with higher doses having stronger effects. TNF-α followed a similar pattern (Figure 6F), indicating that tenuifolin can significantly reduce corticosterone-induced neuroinflammation, with the effect increasing at higher tenuifolin doses.

The present study reveals that ferroptosis plays a pivotal role in stress-induced nerve damage. In the corticosterone-induced neurotoxicity model, the viability of neuronal cells was significantly suppressed, and the expression of ferroptosis-related proteins, such as SLC7A11, GPX4, and Nrf2, was markedly reduced. The addition of tenuifolin mitigated this trend. Concurrently, this study found that corticosterone induced structural changes in the SCL7A11/SCL3A2 complex, which may greatly affect the progression of ferroptosis, and tenuifolin alleviated this phenomenon. Similarly, corticosterone significantly exacerbated oxidative stress and neuroinflammation levels, and tenuifolin retarded those changes. It is noteworthy that oxidative stress and neuroinflammation often occur concurrently with ferroptosis and show a promoting effect on ferroptosis. The results indicate that ferroptosis is deeply involved in neuronal homeostasis, and its excessive development will significantly promote neuronal damage.

## 4. Conclusions

Our results underscore the therapeutic efficacy of PT and its bioactive component, tenuifolin, in mitigating the adverse outcomes of stress-induced nerve damage. For the first time in this study, we found both PT and tenuifolin exerted protective effects against corticosterone-evoked nerve damage. Tenuifolin exerted this protective effect by modulating ferroptosis, neuroinflammation, and oxidative stress, which has never been reported in other studies. Tenuifolin demonstrates a capacity to recalibrate the expression of proteins critical to ferroptosis, including SLC7A11, GPX4, and Nrf2, which were previously perturbed by corticosterone exposure. The present study also innovatively explored the intervention mechanism of tenuifolin from the perspective of structural changes in the SLC7A11/SLC3A2 complex and found it also restored the conformation and binding energy changes in the SLC7A11/SLC3A2 complex induced by corticosterone. Tenuifolin reduced neuroinflammatory and oxidative stress responses, further supporting its neuroprotective potential.

These findings emphasized the ability of tenuifolin to influence ferroptosis, oxidative stress, and neuroinflammation, and then to alleviate corticosterone-evoked nerve damage. Furthermore, ferroptosis-related paths may present a novel therapeutic avenue for treating stress-related nerve damage.

It is worth noting that this study only explored the neuroprotective effects of tenuifolin in cell models. This effect and potential mechanisms have not yet been verified with in vivo models. Since functional components undergo digestion, absorption, metabolism, etc., after consumption, it is ambiguous whether tenuifolin will still exert neuroprotective effects by regulating ferroptosis-related pathways in animals, and further validation is needed. Additionally, this study indicates that the neuroprotective efficacy of tenuifolin could be enhanced by improving the delivery method, given that tenuifolin exhibits relatively low bioavailability.

## Data Availability

The original contributions presented in the study are included in the article, further inquiries can be directed to the corresponding authors.
